# Holothuroidea Have Spongy Bodies Homologous to Spongy Bodies of Echinoidea and Tiedemann’s Bodies of Asteroidea

**DOI:** 10.1134/S0012496625600381

**Published:** 2025-12-02

**Authors:** O. V. Ezhova, V. V. Malakhov

**Affiliations:** Biological Faculty, Moscow State University, Moscow, Russia

**Keywords:** Echinodermata, interradii, tentacles, sea cucumbers, sea urchins, sea stars

## Abstract

Organs that can be considered as homologous to the spongy bodies of sea urchins and Tiedemann’s bodies of sea stars were found in the holothuroid *Chiridota laevis* (O. Fabricius, 1780). The *C. laevis* spongy bodies occur in interradii and are formed by outgrowths of coelomic canals, which connect the water ring with the coelomic cavities of tentacles. Haemocoelic lacunae are adjacent to the coelomic outgrowths. The spongy bodies of sea urchins and Tiedemann’s bodies of sea stars are also in the interradii and indicate the position of reduced tentacles. The spongy bodies were assumed to function as excretory organs (additional kidneys) in echinoderms. Because the water vascular system does not open outwards in most holothuroids, the holothuroid spongy bodies are much smaller than in other echinoderms and can be considered as rudimentary organs.

The phylum Echinodermata includes exclusively marine invertebrates with a unique anatomical organization. Secondary radial symmetry and a water vascular (ambulacral) system are features of echinoderms. The water vascular system derives from the second left coelom of the larva [1]. The system includes a circumoral ring and five radial canals, which branch off the ring and extend into ambulacral podia of echinoderms. Peculiar organs located on the ambulacral ring have been described in sea urchins and sea stars and are known as the spongy bodies (in Echinoidea) [2–4] and Tiedemann’s bodies (in Asteroidea) [3, 5, 6]. The organs occur in interradii and consist of interwoven thin coelomic canals and hemocoelic lacunae in both of the classes.

Organs that are possible to consider as homologs of the spongy bodies of sea urchins and Tiedemann’s bodies of sear stars were found in the holothuroid *Chiridota laevis* (O. Fabricius, 1780) of the order Apodida in a microscopic anatomical study.

We examined three *C. laevis* individuals, which were collected from sandy and silty grounds at depths of 4–40 m by trawling in 2025; the collection site was near the White Sea Biological Station (Moscow State University) in the Kandalaksha Gulf of the White Sea. The body length of the holothuroids ranged from 1.5 to 6 cm. The holothuroids were relaxed in a cold milieu by gradually adding ethanol for 24 h, fixed with 4% formaldehyde, and stored in 70% ethanol after fixation. For a histology study, holothuroid fragments were decalcified with 7% nitric acid according to a standard protocol [7], dehydrated with increasing alcohol concentrations, embedded in paraplast, and used to obtain series of sagittal and transversal sections (5 µm) with a Leica RM 2125RTS microtome (Leica Biosystems, Germany). The sections were stained with Carracci hematoxylin and examined under a Mikmed-6 microscope (LOMO, St. Petersburg, Russia, 2018) with a MS-12 digital camera.

The *C. laevis* spongy bodies occur in the interradii at the bases of the canals that connect the coelomic cavities of the tentacles with the ambulacral ring (Fig. 1a). The canal forms coelomic outgrowths with hemocoelic lacunae between them at the base of a tentacle (Fig. 1b). The wall of a coelomic outgrowth within a spongy body consists of a flattened epithelium of approximately 5 µm in height and lacks a muscle layer, which underlies the coelomic epithelium in the tentacles. A single *C. laevis* sponge body is approximately 50 µm in diameter.

**Fig. 1.**
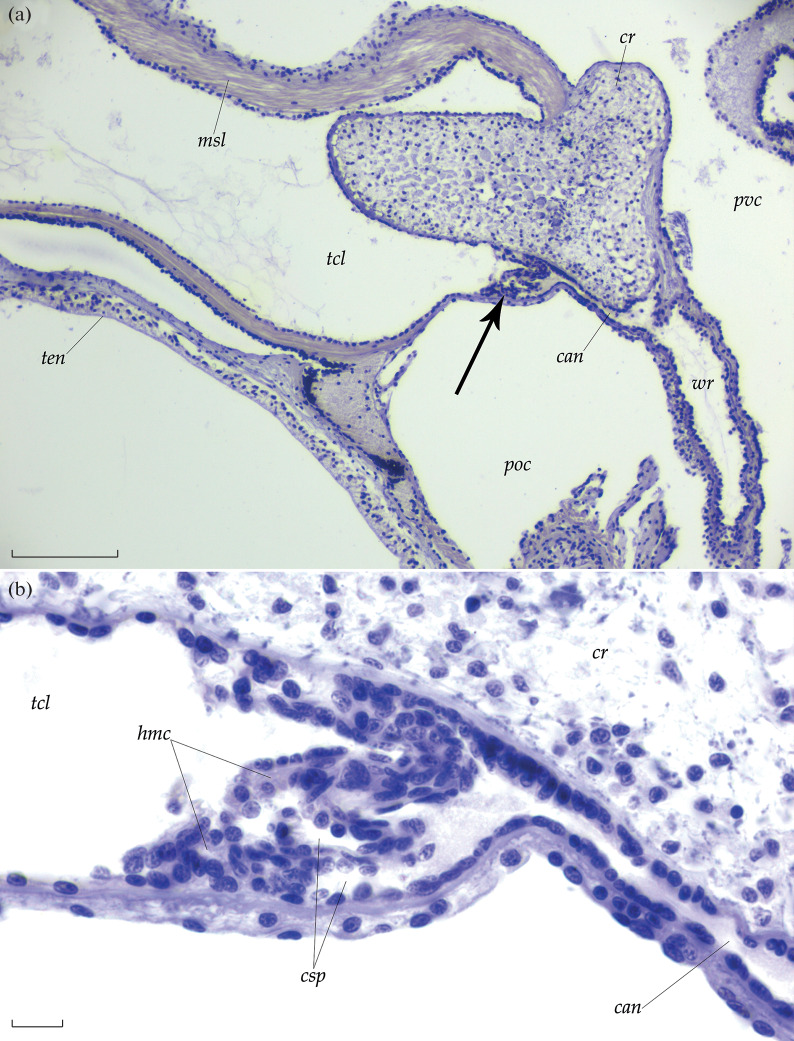
Spongy bodies of the holothuroid *C. laevis*: (а) position of a spongy body (an arrow) at the base of a tentacle and (b) histological structure of the spongy body. Bar, (a) 100 or (b) 10 μm. Designations: *can,* canal connecting the water ring (*wr*) with the tentacular coelom (*tcl*); *cr*, calcareous ring; *csp*, coelomic spaces of the spongy body; *hmc*, hemocoelic lacunae of the spongy body; *msl*, tentacular muscles; *poc*, perioral coelom; *pvc*, perivisceral coelom; *tcl*, coelomic cavity of the tentacle; *ten*, tentacle; *wr*, water ring.

By their position in the interradii, the holothuroid *C. laevis* spongy bodies correspond to the spongy bodies of sea urchins and Tiedemann’s bodies of sea stars (Fig. 2). The *C. laevis* spongy bodies are substantially smaller than their sea urchin and sea star counterparts, which reach 100–120 µm in diameter in individuals comparable in size with the *C. laevis* individuals examined in this work [4–6]. Still the internal structure of the *C. laevis* spongy bodies is similar in organization to the spongy bodies of sea urchins and Tiedemann’s bodies of sea stars, representing a mesh of interwoven coelomic outgrowths and hemocoelic lacunae. Thus, the spongy bodies of the holothuroid *C. laevis* are homologs of the spongy bodies of sea urchins and Tiedemann’s bodies of sea stars according to the first and second of Remane’s criteria for homology, namely, the relative position and special qualities criteria [8].

**Fig. 2.**
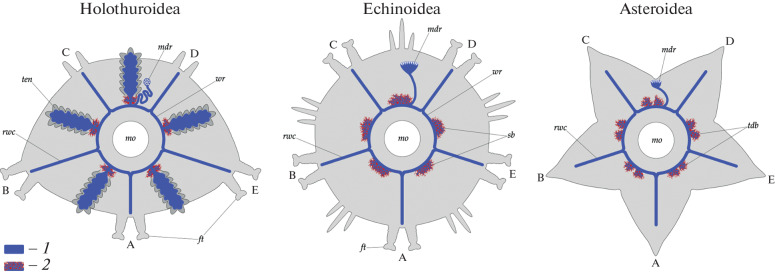
Position of the spongy bodies in relation to the water vascular system in sea cucumbers (Holothuroidea), sea urchins (Echinoidea), and sea stars (Asteroidea). The radii are indicated with capital letters. Designations: *1,* coelom of the water vascular system and tentacles; *2,* spongy bodies; *ft*, tube feet (podia); *mdr*, madreporic plate; *rwc*, radial water vascular canals; *sb*, spongy bodies of echinoids; *tdb*, Tiedemann’s bodies of asteroids; *ten*, tentacles of holothuroids; *wr,* water ring.

Holothuroids are the only echinoderm class where tentacles are preserved. Tentacular coeloms of holothuroids derive from the second left coelom of larvae and are therefore possible to consider as homologs of the left half of the tentacular system of Graptolithoidea (Pterobranchia) [9, 10] and, on a broader scale, the tentacles that develop on the second (perioral) segment in other evolutionary branches of Triploblastica [11, 12]. We think that sea urchins and sea stars have lost tentacles, but preserved the spongy bodies, which indicate the positions of reduced tentacles on the ambulacral ring (Fig. 2).

The histological structure of the spongy organs, which are a mesh of interwoven coelomic outgrowths and hemocoelic lacunae, is similar to the structure of the axial organ of echinoderms [13–15]. The axial organ performs fluid ultrafiltration from the hemocoel through the basal lamina and diaphragms between coelomic lining cells into the axial coelom, wherefrom the urine is then expelled *via* the madreporite. Thus, the axial organ acts as a main kidney in echinoderms [15]. The spongy bodies and Tiedeman’s bodies are likely to perform the excretory function as well and can therefore be considered as additional kidneys. The urine produced in these organs passes through the ambulacral ring and stone canal to reach the madreporite and is expelled there. The madreporic plate is immersed in the perivisceral coelom in many holothuroids, in contrast to other echinoderms. Only four modern orders of Holothuroidea include species with a stone canal opening to the outside [16–20]. Thus, the class Holothuroidea tends to lose the connection between the water vascular system and the environment. The holothuroid spongy bodies are consequently far smaller in size than in other echinoderms and can be considered as rudimentary organs.

## References

[1] Ivanova-Kazas, O.M., *Sravnitel’naya embriologiya bespozvonochnykh zhivotnykh* (Comparative Embryology of Invertebrates), Moscow: Nauka, 1978, vol. 3.

[2] Perrier, E., L’appareil circulatoire des oursins, *Arch. Zool. Exp. Génet.*, 1875, Ser. 2, vol. 4, pp. 605–643.

[3] Hyman, L.H., *The Invertebrates*, vol. 4: *Echinodermata*, New York: McGraw-Hill Book Company, 1955.

[4] Ezhova, O.V., Malakhov, V.V., and Egorova, E.A., Axial complex and associated structures of the sea urchin *Strongylocentrotus pallidus* (Sars, G.O. 1871) (Echinodermata: Echinoidea), *J. Morphol.*, 2018, vol. 279, no. 6, pp. 792–808. 29528140 10.1002/jmor.20811

[5] Hayashi, R., Anatomy of *Henricia sanguinolenta, J. Fac. Sci. Hokkaido Univ.*, 1935, Ser. 6, vol. 4, no. 4, pp. 1–26.

[6] Cuénot, L., Étude anatomique des astérides, *Arch. Zool. Exp. Génet.*, 1887, Ser. 2, vol. 5, pp. 1–144.

[7] Valovaya, M.A. and Kavtaradze, D.N., *Mikrotekhnika: Pravila, priemy, iskusstvo, eksperiment* (Microtechnique: Rules, Maneuvers, Craft, Experiment), Moscow: Mosk. Gos. Univ., 1993.

[8] Remane, A., *Die Grundlagen des Natürlichen Systems der Vergleichenden Anatomie und der Phylogenetik*, Leipzig, 1952.

[9] Fedotov, D.M., To the problem of homology of coeloms of Echinodermata, Enteropneusta and Chordata, *Izv. Biol. Nauchno-Issled. Inst., Perm.**Univ.*, 1923, vol. 2, no. 1, pp. 1–11.

[10] Beklemishev, V.N., *Osnovy sravnitel’noi anatomii bespozvonochnykh* (Fundamentals of Comparative Anatomy of Invertebrates), Moscow: Nauka, 1964, vol. 1.

[11] Malakhov, V.V. and Gantsevich, M.M., The origin and main trends in the evolution of bilaterally symmetrical animals, *Paleontol. J.*, 2022, vol. 56, pp. 887–937.

[12] Malakhov, V.V. and Ezhova, O.V., On the origin of tentacles and limbs in Deuterostomia, *Russ. J. Mar. Biol.*, 2023, vol. 49, suppl. 1, pp. 2–28.

[13] Welsch, U. and Rehkamper, G., Podocytes in the axial organ of echinoderms, *J. Zool. London*, 1987, vol. 213, pp. 45–50.

[14] Ziegler, A., Faber, C., and Bartolomaeus, T., Comparative morphology of the axial complex and interdependence of internal organ systems in sea urchins (Echinodermata: Echinoidea), *Front. Zool.*, 2009, vol. 6, p. 10. 10.1186/1742-9994-6-10 PMC2701938 19508706

[15] Ezhova, O.V. and Malakhov, V.V., The axial complex of echinoderms represents the kidney and is homologous to the hemichordate heart-kidney, *Paleontol. J.*, 2021, vol. 55, no. 9, pp. 1029–1038.

[16] Lastochkin, D.A., On the anatomy and physiology of synaptids (Sinaptidae), *Izv. Imp. S.-Peterb. O-va.**Nat.*, 1914, vol. 45, nos. 1–2, pp. 23–34.

[17] Erber, W., Der Steinkanal der Holothurien: Eine morphologische Studie zum Problem der Protocoelampulle, *Z. Zool. Syst. Evolutionsforsch.*, 1983, vol. 21, no. 3, pp. 217–234.

[18] Lang, A., *Lehrbuch der Vergleichenden Anatomie*, Jena: Verlag von Gustav Fisher, 1894, vol. XVI, p. 1198.

[19] Lang, A., *Text-Book of Comparative Anatomy*, London, New York: MacMilan and Co, 1896, vol. XVI, part II, p. 618.

[20] Smirnov, A.V., Origin of the Class Holothuroidea, *Paleontol. J.*, 2021, vol. 55, no. 7, pp. 766–786

